# Antifibrotic Effect of the TGF-β Type I Receptor Inhibitor EW-7197 on Anastomotic Healing in a Rat Choledochojejunostomy Model

**DOI:** 10.3390/biomedicines14030698

**Published:** 2026-03-17

**Authors:** Fırat Aslan, Serhat Binici, Iklil Eryılmaz, Burhan Beger, Orhan Beger, Ümit Haluk İliklerden, İbrahim Özalp, Zehra Akman İlik, Feyruz Karakoyun, Şahin Şahinalp, Muzaffer Önder Öner, Mehmet Çetin Kotan

**Affiliations:** 1 General Surgery Department, Van Yüzüncü Yıl University, Van 65090, Türkiye; drserhatbinici@gmail.com (S.B.); umithalukiliklerden@hotmail.com (Ü.H.İ.); cetinkotan@hotmail.com (M.Ç.K.); 2 General Surgery Department, Kartal Dr. Lutfi Kirdar City Hospital, İstanbul Provincial Directorate of Health, İstanbul 34865, Türkiye; iklileryilmaz@hotmail.com; 3 Pediatric Surgery Department, Van Yüzüncü Yıl University, Van 65090, Türkiye; burhanbeger@hotmail.com; 4 Department of Anatomy, Faculty of Medicine, Gaziantep University, Gaziantep 27650, Türkiye; obeger@gmail.com; 5 General Surgery Department, Akdamar Hospital, Van 65040, Türkiye; dr.ibrahimozalp@hotmail.com; 6 Pathology Departmant, Van Yüzüncü Yıl University, Van 65090, Türkiye; drz_akman@hotmail.com (Z.A.İ.); feyruzkarakoyun@gmail.com (F.K.); 7 Cardiovascular Surgery Department, Van Yüzüncü Yıl University, Van 65090, Türkiye; ssahinalp@gmail.com; 8 General Surgery Department, Nişantaşı Üniversitesi Bht Clinic, Istanbul 34307, Türkiye; zkudrooner@gmail.com

**Keywords:** vactosertib, EW-7197, biliary anastomosis, fibrosis, rat, choledochojejunostomy, TGF-β

## Abstract

**Background/Aim**: Anastomotic stricture following choledochojejunostomy (CJS) is largely driven by fibrotic remodeling at the anastomotic site, a process mediated by transforming growth factor-β (TGF-β) signaling. This problem is particularly relevant in emergency biliary surgery, where CJS is frequently performed under suboptimal conditions and anastomotic leakage is common, predisposing to exaggerated fibrosis and late strictures. This study aimed to evaluate the effect of the TGF-β type I receptor (ALK5) inhibitor EW-7197 (vactosertib) on histopathological parameters of anastomotic healing, with a particular focus on fibrosis, in a rat CJS model. **Materials and Methods**: Twenty-four male Wistar Albino rats were randomized into three groups (*n* = 8 each): control (G1), CJS only (G2), and CJS plus EW-7197 (G3). EW-7197 was administered as a single intraperitoneal dose (20 mg/kg) immediately after completion of the anastomosis. On postoperative day 21, choledochojejunal anastomotic tissues were harvested and evaluated histologically using hematoxylin–eosin and Masson’s trichrome staining. Edema, hyperemia, inflammation, and fibrosis were graded using a semi-quantitative scoring system, and intergroup comparisons were performed using non-parametric statistical tests. **Results**: Compared with surgery alone, EW-7197 treatment resulted in a statistically significant reduction in fibrosis severity at the anastomotic site (*p* < 0.001) and a significant attenuation of hyperemia (*p* = 0.007). Edema scores showed a downward trend in the EW-7197-treated group but did not reach statistical significance, while inflammation scores did not differ significantly between the surgical groups. **Conclusions**: In this experimental rat choledochojejunostomy model, administration of the selective ALK5 inhibitor EW-7197 significantly reduced histopathological fibrosis and hyperemia at the anastomotic site on postoperative day 21 without affecting inflammation severity. These findings support the role of the TGF-β/Smad pathway in bilioenteric anastomotic fibrotic remodeling. However, further studies including molecular validation and functional assessments are required to clarify the translational relevance of these results.

## 1. Introduction

Choledochojejunostomy (CJS) is a widely used reconstructive procedure for restoring bile–enteric continuity in patients with pancreatic head carcinoma, cholangiocarcinoma, periampullary tumors, and benign biliary strictures, and is considered the gold-standard surgical approach in these settings [1,2]. Despite its effectiveness, CJS is associated with several well-recognized complications, including reflux cholangitis due to bypassing the sphincter of Oddi, bacterial and enterokinase reflux, and progressive fibrotic remodeling at the anastomotic site [3]. Clinical series have reported anastomotic stricture rates ranging from 13% to 58% within the first postoperative year, highlighting the substantial morbidity associated with this procedure despite advances in surgical technique [4,5,6,7]. Consequently, preventing excessive fibrotic remodeling and subsequent anastomotic stricture formation remains a major clinical challenge.

The pathophysiological cascade underlying anastomotic stricture formation begins with early inflammatory responses triggered by exposure of the biliary epithelium to intestinal contents, followed by fibroblast activation, myofibroblast differentiation, increased extracellular matrix (ECM) deposition, and smooth muscle hyperplasia [8]. Transforming growth factor-β (TGF-β) is a central regulator of this inflammatory–fibrotic axis and plays a pivotal role in scar formation by inducing ECM protein synthesis through both Smad-dependent and Smad-independent signaling pathways [9,10,11]. Increased TGF-β expression has been demonstrated in both experimental and clinical studies of biliary tract fibrosis and gastrointestinal anastomotic healing, underscoring its key role in fibrotic remodeling processes [10,11].

Current treatment options for established biliary enteric anastomotic strictures—including balloon dilation, serial stenting, and surgical revision—are associated with recurrence rates exceeding 20% and are often technically challenging due to altered postoperative anatomy [12]. These limitations emphasize the need for preventive strategies aimed at modulating the fibrotic response during the early postoperative healing phase, rather than treating strictures once they have fully developed.

Beyond its profibrotic effects, TGF-β exerts complex immunoregulatory functions by suppressing effector T-cell differentiation while promoting regulatory T-cell (Treg), Th9, and Th17 lineage development, and by influencing B-cell antibody production and class switching [13]. Consequently, several therapeutic strategies targeting TGF-β signaling—such as small-molecule receptor kinase inhibitors, monoclonal antibodies, ligand traps, and bifunctional agents—are currently under investigation, particularly in oncology, where they have demonstrated promising results when combined with immunotherapy [14,15].

EW-7197 (vactosertib) is a next-generation, highly selective inhibitor of the TGF-β type I receptor kinase (ALK5) that has shown potent antifibrotic effects in preclinical models of pulmonary, renal, hepatic, and esophageal fibrosis through inhibition of Smad2/3 phosphorylation and downstream collagen deposition [16,17,18]. Although vactosertib is orally bioavailable, its favorable safety profile and robust antifibrotic activity make it an attractive candidate for investigating postoperative fibrostenotic processes. However, its effects on bile–enteric anastomotic healing and fibrosis have not previously been evaluated.

In addition to elective and oncologic indications, choledochojejunostomy is frequently performed in emergency surgical practice, particularly following iatrogenic bile duct injuries, traumatic biliary disruptions, uncontrolled biliary leakage, or septic abdominal explorations. In such emergency settings, anastomotic healing is often compromised by severe local inflammation, tissue edema, ischemia, and contamination, resulting in an increased risk of anastomotic leakage. Importantly, anastomotic leakage not only contributes to early postoperative morbidity and mortality but also serves as a critical trigger for exaggerated fibrotic remodeling, ultimately predisposing patients to late biliary strictures. From this clinical standpoint, pharmacologic modulation of fibrogenic signaling pathways during the postoperative healing phase may represent a particularly relevant preventive strategy in emergency biliary surgery, where optimal local conditions for anastomotic repair cannot always be ensured.

Experimental investigation of antifibrotic interventions in bile–enteric anastomoses has been limited by the lack of well-characterized small-animal models. While large-animal CJS models are costly and technically demanding, recent studies have demonstrated that a reliable rat Roux-en-Y choledochojejunostomy model with acceptable morbidity and mortality can be established [19]. However, the application of this model to evaluate pharmacologic inhibition of fibrotic signaling pathways remains insufficiently explored.

Accordingly, the present study was designed to evaluate the effect of the TGF-β/ALK5 inhibitor EW-7197 on histopathological parameters of anastomotic healing, including fibrosis, edema, hyperemia, and inflammation, in a rat choledochojejunostomy model. Rather than focusing on established strictures and motivated in part by the high clinical burden of anastomotic leakage and impaired healing encountered in emergency biliary surgery, this study aimed to determine whether targeted inhibition of TGF-β signaling could attenuate fibrotic remodeling at the anastomotic site during the postoperative healing period. The findings of this study may provide an experimental basis for future preventive strategies applicable to both elective and emergency biliary surgery.

## 2. Materials and Methods

### 2.1. Animals

Twenty-four male, 3-month-old Wistar Albino rats, weighing 250–290 g, with no prior experimental manipulation, were included in the study. All animals were housed under standard laboratory conditions with ad libitum access to pellet chow and tap water, maintained on a 12-h light/dark cycle (21 ± 1 °C, 40–70% humidity). Only male rats were used to minimize hormonal variability, and animals were derived from in-house breeding with at least three generations obtained.

### 2.2. Study Design

Rats were randomly assigned to three groups (*n* = 8 per group) using a simple randomization method. Tails were labeled with the group designation and sequence number for identification.

- Group 1 (G1, control group): No surgical or pharmacologic intervention.
- Group 2 (G2, surgery group): Rats underwent choledochojejunostomy (CJS) using a standardized surgical protocol.
- Group 3 (G3, surgery + TGF-β inhibitor group): Rats underwent CJS followed by administration of the TGF-β type I receptor inhibitor EW-7197.

Across the study, a total of 24 rats were used. All animals were sacrificed on postoperative day 21, and choledochojejunal tissues encompassing the anastomotic site were harvested for histopathological evaluation. Histological parameters assessed included fibrosis, inflammation, edema, and hyperemia, which were subsequently analyzed statistically.

The primary endpoint of the study was histopathological assessment of fibrosis at the anastomotic site. Functional parameters such as anastomotic patency, biliary drainage, serum cholestatic markers, duct diameter measurements, or radiologic evaluation of biliary flow were not included in the original experimental design.

### 2.3. TGF-β Inhibitor Administration

Vactosertib (EW-7197; TEW-7197) was administered as a single intraperitoneal dose of 20 mg/kg via the right upper quadrant immediately after completion of the anastomosis. No additional doses or alternative administration routes were evaluated.

The intraperitoneal route was selected to ensure rapid systemic bioavailability and reproducible dosing in small animals. The administered dose (20 mg/kg) was determined based on previously published preclinical fibrosis models demonstrating effective inhibition of the TGF-β/ALK5 pathway [16,17,18]. Although vactosertib is orally bioavailable, repeated dosing schedules, dose–response analyses, or delayed administration strategies were not evaluated in this proof-of-concept study.

### 2.4. Surgical Procedure

Anesthesia was induced using intraperitoneal ketamine (Ketalar^®^, Parke-Davis & Co. Inc., Shanghai, China 75 mg/kg) and xylazine (Rompun^®^, Bayer, Leverkusen, Germany, 10 mg/kg). Following midline laparotomy, the common bile duct was transected at the mid-distal level, and bile flow was confirmed. The distal bile duct was ligated and left in situ. A jejunal loop was mobilized, and an end-to-side choledochojejunostomy was performed to the proximal bile duct using interrupted 7-0 non-absorbable monofilament polypropylene sutures (Ethicon Prolene^®^, Ethicon Inc., Somerville, NJ, USA).

**Figure 1:**
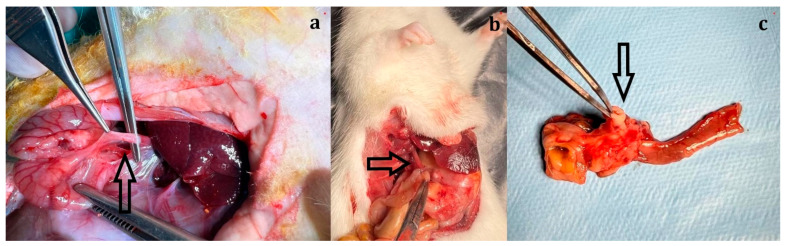
Intraoperative common bile duct (**a**), choledochojejunostomy (**b**), and the specimen excised together with the surrounding tissue after the rat was sacrificed, to be submitted for pathology (**c**) (marked with black arrows).

The abdominal wall was closed using 4-0 silk sutures (DOĞSAN Surgical Silk, 4/0, 18 mm half-circle, Doğsan Tıbbi Malzeme Sanayi A.Ş., Trabzon, Turkey). On postoperative day 21, rats were sacrificed under high-dose anesthesia. Tissues surrounding the choledochojejunal anastomosis were excised and submitted for histopathological examination (Figure 1).

### 2.5. Ethics Statement

This study was approved by the Local Animal Experiments Ethics Committee of Van Yüzüncü Yıl University (approval no: 2022/03-13). All procedures were conducted in accordance with institutional and national guidelines for the care and use of laboratory animals.

### 2.6. Histopathological Evaluation

Excised specimens were fixed in 10% neutral-buffered formalin for 24 h, embedded in paraffin, and sectioned at 4 µm thickness. Sections were stained with hematoxylin–eosin (H&E) for general morphology and Masson’s trichrome for evaluation of collagen deposition. All histopathological assessments were performed by experienced pathologists blinded to group allocation using an Olympus BX53F light microscope.

**Figure 2:**
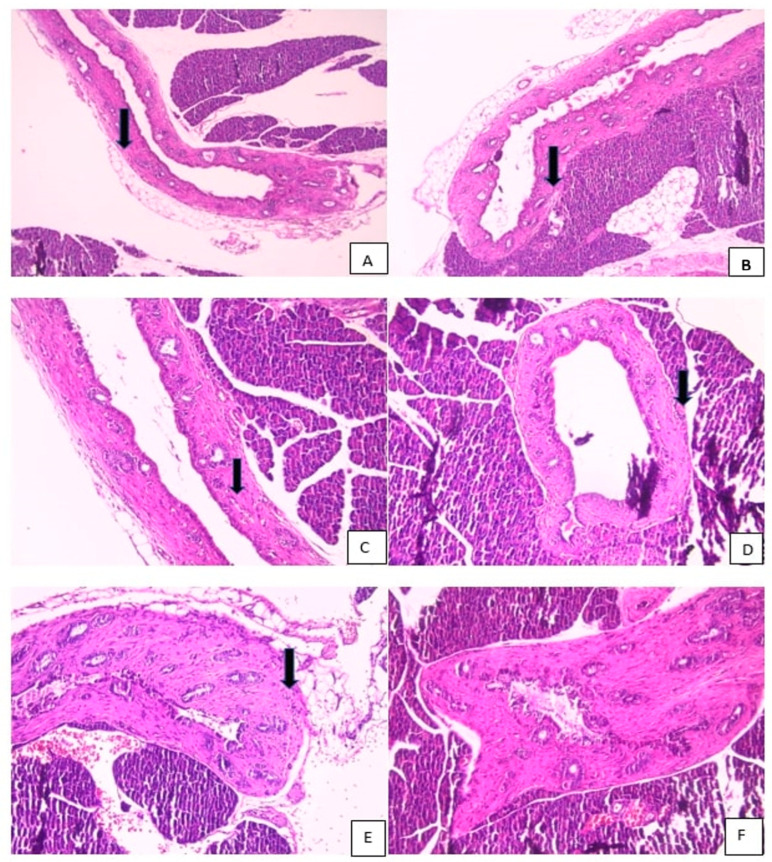
(**A**) Common bile duct tissue with near-normal histological features (black arrow: minimal hyperemia) (H&E ×100). (**B**) Common bile duct tissue with near-normal histological features (black arrow: minimal lymphocytic inflammation) (H&E ×100). (**C**) Common bile duct tissue showing moderate hyperemia (black arrow) (H&E ×100). (**D**) Common bile duct tissue with mild hyperemia and moderate lymphocytic inflammation (black arrow) (H&E ×100). (**E**) Common bile duct tissue showing moderate lymphocytic inflammation (black arrow) and moderate fibrosis (H&E ×200). (**F**) Common bile duct tissue exhibiting marked fibrosis (H&E ×200).

**Figure 3:**
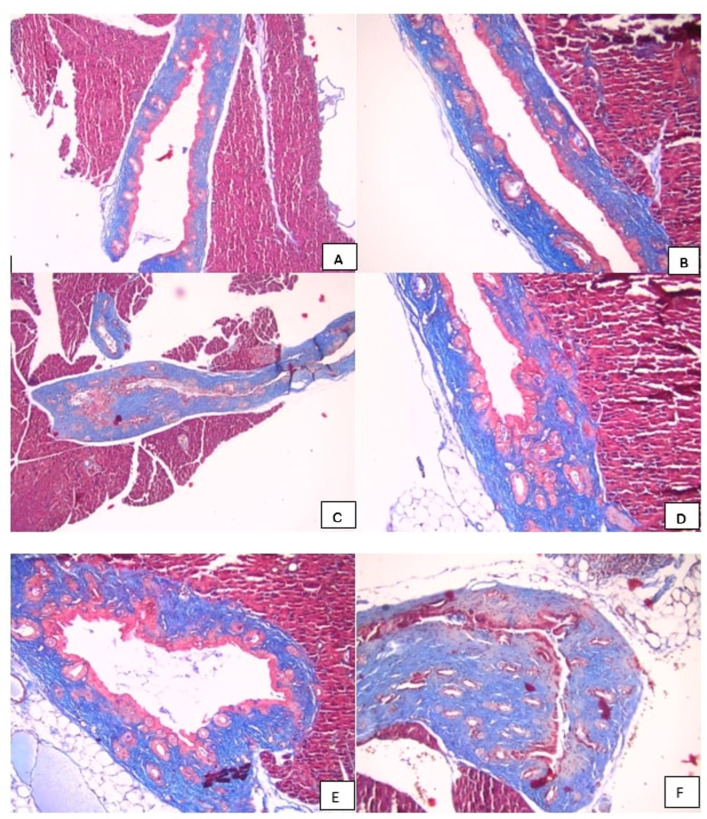
(**A**) Nearly normal common bile duct tissue (Masson’s trichrome, ×100). (**B**) Nearly normal common bile duct tissue (Masson’s trichrome, ×100). (**C**) Mild fibrosis in the wall of the common bile duct (Masson’s trichrome, ×100). (**D**) Mild fibrosis in the wall of the common bile duct (Masson’s trichrome, ×100). (**E**) Moderate fibrosis in the wall of the common bile duct (Masson’s trichrome, ×100). (**F**) Marked fibrosis in the wall of the common bile duct (Masson’s trichrome, ×200).

**Figure 4:**
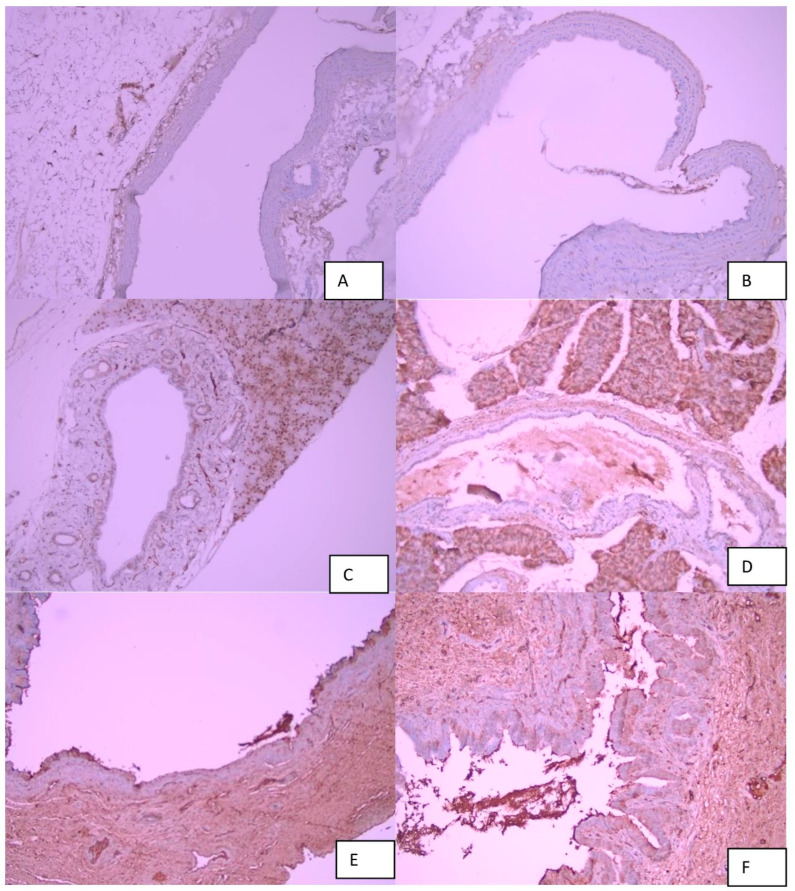
(**A**) Normal-appearing bile duct tissue (TGF β ×100). (**B**) Normal-appearing bile duct tissue (TGF β ×100). (**C**) Mild fibrosis in the bile duct wall (TGF β ×100). (**D**) Mild fibrosis in the bile duct wall (TGF β ×100). (**E**) Marked fibrosis in the bile duct wall (TGF β ×100). (**F**) Moderate fibrosis in the bile duct wall (TGF β ×200).

Edema, hyperemia, inflammation, and fibrosis were scored semi-quantitatively as absent (−), mild (+), moderate (++), or severe (+++), based on a modified scoring system described by Taşdemir et al. [20]. Score-based analyses were used for all intergroup statistical comparisons (Figure 2, Figure 3 and Figure 4).

The semi-quantitative scoring criteria were defined as follows:

Fibrosis:

(−) No detectable collagen deposition;

(+) Mild collagen deposition involving less than 25% of the bile duct wall or perianastomotic tissue;

(++) Moderate collagen deposition involving 25–50% of the wall thickness;

(+++) Dense collagen deposition involving more than 50% of the wall with architectural distortion.

Inflammation:

(−) No inflammatory cell infiltration;

(+) Scattered lymphocytes without structural distortion;

(++) Diffuse inflammatory infiltration with mild architectural alteration;

(+++) Dense transmural inflammatory infiltration with marked structural disruption.

Edema:

(−) Absent;

(+) Mild interstitial expansion;

(++) Moderate interstitial separation of tissue layers;

(+++) Marked tissue expansion with evident separation of structural components.

Hyperemia:

(−) Normal vascular appearance;

(+) Mild vascular congestion;

(++) Moderate vascular dilation and congestion;

(+++) Marked vascular engorgement with prominent luminal dilation.

Scoring was performed independently by two blinded pathologists, and consensus was reached in cases of discrepancy.

### 2.7. Immunohistochemical Analysis for TGF-β

Additional paraffin-embedded sections (4 µm) were subjected to immunohistochemical staining to evaluate TGF-β expression. Following deparaffinization and rehydration, antigen retrieval was performed using citrate buffer (pH 6.0) in a microwave oven. Endogenous peroxidase activity was blocked with 3% hydrogen peroxide. Sections were incubated overnight at 4 °C with a primary anti-TGF-β antibody (rabbit polyclonal, dilution 1:100).

After washing, sections were incubated with a biotinylated secondary antibody and subsequently treated with streptavidin–horseradish peroxidase. Immunoreactivity was visualized using 3,3′-diaminobenzidine (DAB) as the chromogen, and nuclei were counterstained with hematoxylin. Negative controls were prepared by omitting the primary antibody.

TGF-β immunoreactivity was evaluated qualitatively based on staining intensity and distribution within the bile duct wall and perianastomotic tissue, and representative images were recorded for comparison among study groups (Figure 4).

Immunohistochemical evaluation was qualitative in nature and was not subjected to quantitative image analysis or densitometric measurement.

### 2.8. Statistical Analysis

Power analysis was performed using G*Power software (version 3.1.9.2), yielding a total sample size of 24 animals (three groups of eight) based on 80% power, a 5% alpha error, and an effect size of 0.50. Statistical analyses were conducted using IBM SPSS Statistics (version 22.0).

Categorical comparisons of the presence or absence of histopathological findings were analyzed using the chi-square test. Score-based intergroup comparisons were performed using the Kruskal–Wallis test, followed by Mann–Whitney U tests with Bonferroni correction for pairwise comparisons where appropriate. A *p*-value < 0.05 was considered statistically significant.

## 3. Results

### 3.1. Histopathological Evaluations

Histopathological evaluation of choledochojejunal anastomotic tissues was performed using hematoxylin–eosin (H&E) and Masson’s trichrome (MT) staining at ×100 and ×200 magnifications. Specimens were fixed in 10% neutral-buffered formalin, embedded in paraffin, and sectioned at 4 µm thickness. All slides were independently evaluated by experienced pathologists blinded to group allocation. Representative histological images are presented in Figure 2, Figure 3 and Figure 4.

The study groups were compared with respect to edema, hyperemia, fibrosis, and inflammation using both categorical (presence/severity) and score-based semi-quantitative analyses, as summarized in Tables S1 and S2.

### 3.2. Edema Evaluation

#### 3.2.1. Categorical Analysis (Chi-Square Test)

Edema was observed exclusively in the surgical groups (G2 and G3), while no edema was detected in the control group (G1) (*p* < 0.001, Supplemental Table S1). In G2, one rat exhibited moderate edema, and the remaining rats showed mild edema. In G3, all rats demonstrated mild edema.

#### 3.2.2. Score-Based Analysis (Kruskal–Wallis Test)

Edema scores differed significantly among the three groups (H = 21.44, df = 2, *p* < 0.001, ε^2^ = 0.93) (Supplemental Table S2). Post hoc analysis revealed significantly lower edema scores in G1 compared with G2 (*p* < 0.001, *r* = 0.94) and G3 (*p* < 0.001, *r* = 0.97). No statistically significant difference was observed between G2 and G3 (*p* = 0.317).

Accordingly, EW-7197 administration was not associated with a statistically significant reduction in edema severity.

### 3.3. Hyperemia Evaluation

#### 3.3.1. Categorical Analysis (Chi-Square Test)

Hyperemia was present across all groups (*p* = 0.007, Supplemental Table S1). In G1, three rats exhibited no hyperemia, while the remaining animals showed mild hyperemia. In G2, all rats demonstrated hyperemia ranging from mild to severe. In G3, hyperemia was predominantly mild, with only one rat exhibiting moderate hyperemia.

#### 3.3.2. Score-Based Analysis (Kruskal–Wallis Test)

Hyperemia scores differed significantly among the groups (H = 12.78, df = 2, *p* = 0.002, ε^2^ = 0.51) (Supplemental Table S2). Post hoc comparisons showed significantly lower hyperemia scores in G1 compared with G2 (*p* = 0.003, *r* = 0.76) and G3 (*p* = 0.044, *r* = 0.50), and also in G3 compared with G2 (*p* = 0.014, *r* = 0.61).

These findings indicate that EW-7197 treatment was associated with a significant attenuation of hyperemia severity at postoperative day 21.

### 3.4. Fibrosis Evaluation

#### 3.4.1. Categorical Analysis (Chi-Square Test)

Fibrosis was detected in both surgical groups (G2 and G3), while no fibrosis was observed in the control group (*p* < 0.001, Supplemental Table S1). All rats in G2 exhibited severe fibrosis (+++). In contrast, in G3, the majority of rats showed mild fibrosis (+), with only one rat demonstrating moderate fibrosis (++).

#### 3.4.2. Score-Based Analysis (Kruskal–Wallis Test)

Fibrosis scores differed significantly among the three groups (H = 22.69, df = 2, *p* < 0.001, ε^2^ = 0.99) (Supplemental Table S2). Post hoc analysis revealed significantly lower fibrosis scores in G1 compared with G2 (*p* < 0.001, *r* = 0.97) and G3 (*p* < 0.001, *r* = 0.94), and also in G3 compared with G2 (*p* < 0.001, *r* = 0.94).

Thus, EW-7197 administration resulted in a marked and statistically significant reduction in fibrosis severity.

### 3.5. Inflammation Evaluation

#### 3.5.1. Categorical Analysis (Chi-Square Test)

Inflammation was present in all rats in the surgical groups (G2 and G3), while absent in the control group (*p* < 0.001, Supplemental Table S1). Inflammation severity in G2 and G3 ranged from mild to moderate.

#### 3.5.2. Score-Based Analysis (Kruskal–Wallis Test)

Inflammation scores differed significantly among the groups (H = 18.40, df = 2, *p* < 0.001, ε^2^ = 0.78) (Supplemental Table S2). Post hoc comparisons demonstrated significantly lower inflammation scores in G1 compared with G2 and G3 (*p* < 0.001 and *r* = 0.93 for both). No statistically significant difference was observed between G2 and G3 (*p* > 0.05).

Accordingly, no statistically significant difference in inflammation severity was observed between the surgical groups at postoperative day 21.

## 4. Discussion

Transforming growth factor-β (TGF-β) is a central mediator of the fibrostenotic cascade and promotes scar formation by enhancing fibroblast proliferation, myofibroblast transdifferentiation, and type I/III collagen synthesis through Smad2/3-mediated signaling pathways [5,6,7,9,10,11,13]. Accordingly, pharmacologic strategies targeting the TGF-β/ALK5 pathway have emerged as promising approaches for attenuating fibrotic remodeling rather than modulating early inflammatory responses. EW-7197 (vactosertib), a selective TGF-β type I receptor (ALK5) inhibitor, has demonstrated antifibrotic effects in multiple preclinical models—including pulmonary, renal, hepatic, and esophageal fibrosis—primarily by suppressing Smad2/3 phosphorylation and downstream extracellular matrix accumulation [9,10,11,16,17,18,20,21].

To our knowledge, the effect of EW-7197 on bilioenteric anastomotic healing has not previously been investigated. Therefore, the present study expands the preclinical field of application of ALK5 inhibition to gastrointestinal anastomotic fibrosis, specifically in a rat choledochojejunostomy model.

In the present rat choledochojejunostomy model, administration of EW-7197 resulted in a statistically significant reduction in fibrosis severity and hyperemia at the anastomotic site compared with surgery alone (fibrosis, *p* < 0.001; hyperemia, *p* = 0.007). In contrast, edema showed a non-significant downward trend, and inflammation scores did not differ significantly between the surgical groups. These findings are consistent with the established role of TGF-β signaling in driving collagen deposition and scar maturation during gastrointestinal anastomotic healing and support the involvement of this pathway in the pathophysiology of bilioenteric anastomotic fibrosis [6,7,8,9,10,11,21,22,23,24,25,26,27,28].

Importantly, inflammation was assessed at a single late postoperative time point (day 21). Since the peak inflammatory phase following gastrointestinal surgery typically occurs within the first postoperative week, the absence of a difference between groups at day 21 does not exclude potential early-phase immunomodulatory effects of ALK5 inhibition. Rather, the findings suggest that EW-7197 did not significantly alter residual inflammatory activity during the remodeling phase of healing. Future studies incorporating early time points (e.g., postoperative days 3–7) are required to clarify temporal effects on inflammatory dynamics.

The marked attenuation of fibrosis in the EW-7197-treated group represents the most striking finding of this study. While all animals in the surgery-only group exhibited severe fibrosis, the majority of EW-7197-treated rats demonstrated only mild fibrosis, with no cases of severe fibrotic change. This pattern strongly suggests effective interruption of the TGF-β/Smad2/3 axis at the anastomotic site. These results are in agreement with previous reports demonstrating the antifibrotic efficacy of vactosertib across diverse organ systems, including lung, liver, kidney, and radiation-induced fibrosis models [16,17,18,29,30,31,32,33].

Although Smad2/3 phosphorylation was not directly assessed by Western blot analysis in the present study, the central role of the TGF-β/Smad signaling pathway in fibrotic processes has been well established in the literature. Numerous experimental studies have demonstrated that TGF-β1 stimulation leads to increased Smad2/3 phosphorylation, which can be reliably evaluated at the protein level using Western blot techniques, and that this activation is markedly attenuated following antifibrotic or TGF-β receptor-targeted interventions [29,30,34,35]. Therefore, while molecular confirmation was beyond the scope of the present design, the pronounced histopathological reduction in fibrosis is mechanistically consistent with the established molecular effects of ALK5 inhibition reported in prior experimental models.

Edema and hyperemia reflect early microcirculatory responses and increased vascular permeability following surgical trauma. In the present study, EW-7197 treatment was associated with a significant reduction in hyperemia but not edema. Given the known roles of TGF-β in endothelial–stromal interactions and vascular remodeling, selective attenuation of hyperemia without a parallel reduction in edema is biologically plausible [9,28].

From a pharmacologic perspective, the drug was administered as a single intraperitoneal dose immediately after completion of the anastomosis. Intraperitoneal delivery was chosen to achieve rapid systemic absorption and consistent dosing in small animals. However, TGF-β-driven fibrosis is a dynamic and prolonged biological process. It is therefore conceivable that repeated dosing regimens, delayed administration strategies, or oral delivery—given the oral bioavailability of vactosertib—might result in different magnitudes or duration of antifibrotic effects. Dose–response analyses were not performed in the present study and warrant further investigation.

From a clinical standpoint, bilioenteric anastomotic strictures represent a significant long-term complication following biliary reconstruction [5,6,7,12]. However, it should be emphasized that the present experimental model reflects elective, clean surgical conditions rather than emergency or septic scenarios. Emergency biliary surgery was discussed to contextualize potential translational relevance, but no leakage, ischemia, or contamination model was employed. Therefore, extrapolation to emergency settings should be interpreted cautiously and viewed as hypothesis-generating rather than confirmatory.

Taken together, the findings of this study suggest that EW-7197 exerts a predominantly antifibrotic effect during the remodeling phase of bilioenteric anastomotic healing, rather than acting as a primary anti-inflammatory agent. The data support a role for TGF-β/ALK5 signaling in anastomotic fibrotic remodeling at the histopathological level. However, functional outcomes such as luminal patency, biliary drainage, or biochemical markers of cholestasis were not evaluated; thus, the clinical implications of the observed histologic improvements remain to be determined.

Nevertheless, several limitations must be acknowledged. Functional outcomes such as anastomotic patency, biliary flow, or long-term stricture formation were not assessed, and therefore, the clinical implications of the observed histopathological improvements remain speculative. In addition, only a single postoperative time point was evaluated, precluding analysis of early inflammatory dynamics and long-term remodeling. Future studies incorporating functional assessments, multiple time points, dose–response analyses, and molecular markers of fibrosis are warranted to further clarify the translational relevance of TGF-β inhibition in bilioenteric reconstruction.

## 5. Limitations

This study has several important limitations that should be carefully considered when interpreting the findings.

First, histopathological evaluation was performed at a single postoperative time point (day 21). As gastrointestinal anastomotic healing is a dynamic and phase-dependent process—characterized by early inflammation (postoperative days 1–7), followed by proliferation and remodeling—evaluation at a single late time point does not allow assessment of temporal changes in inflammatory or fibrotic responses. Therefore, early immunological alterations potentially influenced by ALK5 inhibition may have been missed.

Second, only histopathological parameters were assessed. Functional outcomes such as anastomotic patency, luminal diameter, biliary drainage, biochemical cholestasis markers, or long-term stricture formation were not evaluated. Consequently, although fibrosis severity was significantly reduced, the direct clinical relevance of this histological improvement remains to be determined.

Third, molecular analyses of the TGF-β/Smad signaling pathway—such as Smad2/3 phosphorylation levels assessed by Western blot or immunohistochemistry—were not performed. Although the antifibrotic effect observed is mechanistically consistent with prior experimental literature on ALK5 inhibition, direct molecular confirmation within the anastomotic tissue was beyond the scope of the present design.

Fourth, EW-7197 was administered as a single intraperitoneal dose immediately after surgery. Dose–response relationships, repeated dosing regimens, delayed treatment protocols, or alternative routes of administration (e.g., oral delivery) were not investigated. Given that fibrosis is a progressive and prolonged biological process, different dosing strategies may yield different magnitudes of antifibrotic effect.

Finally, the experimental model reflects controlled elective surgical conditions in healthy rats and does not simulate emergency, septic, ischemic, or leakage-associated scenarios. Therefore, extrapolation of these findings to complex clinical settings should be approached with caution.

Taken together, while the present study demonstrates a significant reduction in histopathological fibrosis at postoperative day 21 following ALK5 inhibition, further experimental investigations incorporating multiple time points, functional outcome measures, molecular validation, and optimized dosing strategies are required before translational implications can be fully established.

## 6. Conclusions

In this experimental rat choledochojejunostomy model, administration of the selective TGF-β type I receptor (ALK5) inhibitor EW-7197 was associated with a significant reduction in histopathological fibrosis and hyperemia at the anastomotic site at postoperative day 21.

No significant difference in inflammation severity was observed between the surgical groups at this late postoperative time point, suggesting that the primary effect of ALK5 inhibition may be related to modulation of fibrotic remodeling rather than persistent inflammatory activity.

These findings support the involvement of the TGF-β/Smad signaling pathway in bilioenteric anastomotic fibrotic remodeling.

However, given that functional outcomes, molecular signaling markers, and long-term structure formation were not evaluated, the clinical relevance of these histopathological improvements remains to be established.

Further experimental studies incorporating multiple postoperative time points, molecular validation, functional assessments, and optimized dosing strategies are required before translational implications can be fully defined.

## Data Availability

The data can be made available from the authors on request. The data are not publicly available due to data sharing restrictions.

## Supplementary Materials

The following supporting information can be downloaded at: https://www.mdpi.com/article/10.3390/biomedicines14030698/s1, Table S1: Evaluation of the histopathological parameters according to the rat numbers in study groups (Chi-square test); Table S2: Comparison of scores belonging to the histopathological parameters of the study groups (Kruskal–Wallis Test).
