# Supplementary material for: Antifibrotic Effect of the TGF-β Type I Receptor Inhibitor EW-7197 on Anastomotic Healing in a Rat Choledochojejunostomy Model
Source: Biomedicines. 2026 Mar 17;14(3):698. doi: 10.3390/biomedicines14030698 (PMC13024330; doi:10.3390/biomedicines14030698)
Supplement: Supplementary file 1 [file biomedicines-14-00698-s001.zip › biomedicines-4162532-supplementary.pdf]

Supplemental Table S1. Evaluation of the histopathological parameters according to the rat numbers in study groups (Chi-square test).

**Edema**	0 (-)	1 (+)	2 (++)	3 (+++)	Total	*p values*	**Hyperemia**	0 (-)	1 (+)	2 (++)	3 (+++)	Total	*p values*
G1	8 (100%)	0 (0%)	0 (0%)	-	8	< 0.001	G1	3 (37.5%)	5 (62.5%)	0 (0%)	0 (0%)	8	0.007
G2	0 (0%)	7 (87.5%)	1 (12.5%)	-	8		G2	0 (0%)	2 (25%)	5 (62.5%)	1 (12.5%)	8	
G3	0 (0%)	8 (100%)	0 (0%)	-	8		G3	0 (0%)	7 (87.5%)	1 (12.5%)	0 (0%)	8	
Total	8	15	1	-	24		Total	3	14	6	1	24	
**Fibrosis**	0 (-)	1 (+)	2 (++)	3 (+++)	Total	*p values*	**Inflammation**	0 (-)	1 (+)	2 (++)	3 (+++)	Total	*p values*
G1	8 (100%)	0 (0%)	0 (0%)	0 (0%)	8	< 0.001	G1	8 (100%)	0 (0%)	0 (0%)	-	8	< 0.001
G2	0 (0%)	0 (0%)	0 (0%)	8 (100%)	8		G2	0 (0%)	6 (75%)	2 (25%)	-	8	
G3	0 (0%)	7 (87.5%)	1 (12.5%)	0 (0%)	8		G3	0 (0%)	6 (75%)	2 (25%)	-	8	
Total	8	7	1	8	24		Total	8	12	4	-	24	

G1: the control group, G2: the surgical group, G3: the surgical + TGF-β group, 0 (-): absent, 1 (+): mild, 2 (++): moderate, 3 (+++): severe.

Supplemental Table S2. Comparison of scores belonging to the histopathological parameters of the study groups (Kruskal-Wallis Test).

**Parameters**	**G1**	**G2**	**G3**	***p values***
Edema	0 (0-0)$^{a, b}$	1 (1-2)	1 (1-1)	< 0.001
Hyperemia	1 (0-1)$^{a, b}$	2 (1-3)^{b}	1 (1-2)	0.002
Fibrosis	0 (0-0)$^{a, b}$	3 (3-3)^{b}	1 (1-2)	< 0.001
Inflammation	0 (0-0)$^{a, b}$	1 (1-2)	1 (1-2)	< 0.001

Parameters are presented in the table as median (minimum-maximum). G1: the control group, G2: the surgical group, G3: the surgical + TGF-β group, ^{a}: comparison to G2, ^{b}: comparison to G3, $p < 0.05$.
